# Deposition of Ultrathin Nano-Hydroxyapatite Films on Laser Micro-Textured Titanium Surfaces to Prepare a Multiscale Surface Topography for Improved Surface Wettability/Energy

**DOI:** 10.3390/ma9110862

**Published:** 2016-10-25

**Authors:** Maria Surmeneva, Polina Nikityuk, Michael Hans, Roman Surmenev

**Affiliations:** 1 Department of Experimental Physics, National Research Tomsk Polytechnic University, Lenin Avenue 30, Tomsk 634029, Russia; feja-mari@yandex.ru (M.S.); baitiane@list.ru (P.N.); 2 Functional Materials, Materials Science Department, Saarland University, Saarbrücken 66123, Germany; mail.michaelhans@web.de

**Keywords:** biocompatible coating, hydroxyapatite, multiscale topography, rf magnetron sputtering, surface patterning

## Abstract

The primary aim of this study was to analyse the correlation between topographical features and chemical composition with the changes in wettability and the surface free energy of microstructured titanium (Ti) surfaces. Periodic microscale structures on the surface of Ti substrates were fabricated via direct laser interference patterning (DLIP). Radio-frequency magnetron sputter deposition of ultrathin nanostructured hydroxyapatite (HA) films was used to form an additional nanoscale grain morphology on the microscale-structured Ti surfaces to generate multiscale surface structures. The surface characteristics were evaluated using atomic force microscopy and contact angle and surface free energy measurements. The structure and phase composition of the HA films were investigated using X-ray diffraction. The HA-coated periodic microscale structured Ti substrates exhibited a significantly lower water contact angle and a larger surface free energy compared with the uncoated Ti substrates. Control over the wettability and surface free energy was achieved using Ti substrates structured via the DLIP technique followed by the deposition of a nanostructured HA coating, which resulted in the changes in surface chemistry and the formation of multiscale surface topography on the nano- and microscale.

## 1. Introduction

Currently, a large number of devices and implants are used in medicine [1]. Biomaterials in the form of implants (e.g., ligaments, vascular grafts, heart valves, intraocular lenses, and dental implants) and medical devices (e.g., pacemakers, biosensors, and artificial hearts) are extensively used to replace and/or restore the function of disturbed or deteriorated tissues or organs, and thus improve the quality of life and longevity of human beings [2,3]. The field of biomaterials has shown rapid growth in keeping up with the demands of an aging population [2,3].

Implants should not only be mechanically resistant, but should also be able to rapidly heal the host organism. When implanted into living tissue, all materials initiate a host response, and this represents the first steps of tissue repair [4,5]. Modern implant design is directed towards making use of this immune response to improve implant integration while preventing the perpetuation of the immune response, which leads to chronic inflammation, foreign body reactions, and thus loss of the intended function [4,5]. In addition to determining some of the deformation and strength characteristics of implants (such as the durability, elasticity, and shape stability), it is necessary to minimize the trauma caused by their use and to view the implantation with respect to healing a wound in order to achieve a fast and full postoperative rehabilitation of patients [5]. As a result, it is necessary to fulfil frequently inconsistent requirements with respect to the physical and mechanical properties of the materials used to manufacture the specified products [5]. The physicochemical properties of implants strongly depend on the method used to form the surface of the implants. Numerous techniques have been used to enhance the surface compatibility of tissue implants. Most of these methods involve multiple preparation procedures that incorporate coating and/or patterning steps. Direct laser interference patterning (DLIP) requires a single processing step and can be applied to a wide range of materials. The principle behind this method is based on the unique intensity pattern generated by interfering laser beams, which show periodicities in the (sub-)micron range [6,7,8].

The compatibility of the medical implant surface with biological tissues is achieved through the use of a biocompatible coating, which can be a layer of calcium phosphate (CaP). Biologically-relevant CaP belongs to the orthophosphate group, and naturally occurs in several biological structures, including teeth and bone [9]. Bone consists of an inorganic component of biological apatite as well as an organic component, which primarily consists of collagen and water. Currently, hydroxyapatite (HA), Ca_10_(PO_4_)_6_(OH)_2_, is an optimum material for clinical practice, with a similar structure to that of the mineral component of bone tissue [9]. The basic technological methods that are used to prepare biocompatible coatings include plasma spraying, ion-beam deposition, radio-frequency (rf) magnetron sputtering, and electrochemical deposition. Rf magnetron sputtering is a prospective technique for the fabrication of implant coatings, because it can be used to form HA films that have low roughness and exhibit good adhesion to Ti substrate [10,11]. The plasma parameters of the rf magnetron sputtering process affect the physicochemical and mechanical properties of the CaP films [12]. The deposition parameters can be adjusted to produce single-phase HA films generated at a high deposition rate and high thermal stability. Moreover, the chemical composition of the precursor material used to deposit the HA coating is preserved [13]. The biocompatibility of HA has been thoroughly investigated and established, and it has been shown to promote the proliferation and differentiation of mesenchymal stem cells and adhesion of human keratinocyte cell lines. In addition, the improved adhesion, proliferation, and differentiation, the increased alkaline phosphatase activity of primary human osteoblast cells, and the normal cell growth of human embryonic kidney cell lines in the presence of HA have been shown experimentally [14].

The living tissue and the artificial implant interact at the molecular level, and the size of the biological structures ranges from a few nanometres to tens of micrometres. The scientific experience gained to date has demonstrated that the successful interaction between the biotissue and the surface of the implant frequently depends on physico-chemical material properties, such as the chemical composition, microstructure, roughness, wetting angle, and free surface energy (FSE) [15]. Thus, the aim of this study was to achieve the surface structuring of Ti using the DLIP technique followed by HA coating deposition through rf magnetron sputtering with pure HA. Analyses of the correlation between variations in the topographical multiscale Ti surface feature, including Ti coated with HA, and changes in the surface wettability and energy are reported.

## 2. Materials and Methods

### 2.1. Sample Preparation

Technically pure Ti was used as a substrate. A high-powered pulsed Nd:YAG laser (Quanta-Ray PRO210, Spectra Physics, Santa Clara, CA, USA) was used for laser interference patterning. The repetition rate and pulse duration of the laser were 10 Hz and 10 ns, respectively. The fundamental wavelength of the Nd:YAG laser system was 1064 nm, and shorter wavelengths were obtained through second-harmonic generation. Samples with a surface area of 20 × 20 ${\mathrm{mm}}^{2}$ were irradiated at 355 nm with multiple adjacent 1 × 1–2 × 2 mm^2^ spots at a fluency of 3.05 ± 0.15 J·cm^2^. Line-like structures with periodicities of approximately 4.5 µm and 8.4 µm were obtained using a two-beam laser setup. For cross-like structure types with the same periodicity, samples were structured once, rotated 90°, and then structured a second time.

### 2.2. Coating Deposition

A commercially available apparatus with a rf magnetron source (13.56 MHz) was used to deposit the nanostructured HA coating [12,13]. The HA coating was deposited at an operating pressure of 0.4 Pa (the vacuum chamber was evacuated to 10^−4^ Pa) at a target–substrate distance of 40 mm, with argon as the working gas, and with an rf generator power of 500 W. The HA coating was deposited for 8 h onto a substrate mounted on a grounded substrate holder, which resulted in a coating thickness of 650 ± 50 nm. A target of pure HA—synthesized by the mechanochemical method—was prepared according to the previously described procedures [16,17]. The target for rf magnetron sputtering (220 mm diameter, 10 mm thick) was prepared via ceramic technology—i.e., the powder was pressed at a pressure of 70 MPa and then annealed at 1100 °C for 1 h in air.

### 2.3. Atomic Force Microscopy (AFM) Measurements

The quantitative analysis of the surface morphology of the uncoated and HA-coated substrates was performed with a Solver P47-PRO (NT-MDT, Moscow, Russia) atomic force microscope (AFM) using triangular golden silicon probes (NT-NDT) with a typical spring constant of 28 N·$m^{-1}$ and a resonance frequency of 420 kHz. All of the images were collected in contact AFM mode in air at a typical frequency of 1.5 Hz with an image resolution of 256 points per line. Squares of different sizes (5 × 5 ${\mu m}^{2}$ and 35 × 35 ${\mu m}^{2}$) were scanned, and the Nova SPM software (NT-MDT, Moscow, Russia) was used to analyse the surface roughness. Three different 3D parameters were used to characterize the surface roughness: (*S*_a_), which is the arithmetic mean of the absolute values of the surface departure from the mean plane in the samples area; the root mean square roughness (*S*_q_), which is an index used to represent the standard deviation of the surface heights; and *S*_dr_, which is the developed interfacial area ratio.

### 2.4. X-ray Diffraction (XRD)

An X-ray diffractometer (Shimadzu XRD-6000, Tokyo, Japan) was used to identify the crystalline structure of the HA-coated Ti that were previously treated to prepare parallel and crossed grooves with different periodicities. The typical irradiation conditions were 40 kV and 30 mA using Cu-K_α_ radiation (1.5405 Å); the 2θ scan ranged from 10° to 60° with a step size of 0.02° at a speed of 2 deg/min and a grazing angle of 3°. The average crystallite size was determined using Scherrer’s equation from the broadening of the diffraction peaks; this determination was performed with the Powder Cell 2.4 software (FIMRT, Berlin, Germany), and the instrumental broadening was considered. An instrumental broadening of 0.1° in 2θ was determined by the full width at half maximum (FWHM) of a silicon powder.

### 2.5. Contact Angle and Surface Free Energy Measurements

Contact angle analyses were performed using an optical contact angle apparatus (OCA15 Plus Data Physics Instruments GmbH, Filderstadt, Germany) along with the SCA20 software (Data Physics Instruments GmbH, Filderstadt, Germany). The contact angle (CA) of water in air was measured using a sessile drop method. A minimum of 10 droplets (2 μL, 5 μL$\cdot s^{-1}$) of water and three droplets of diiodomethane or ethylene glycol were seeded on the surface of each sample. The surface free energy was calculated using the Owens–Wendt–Rabel–Kaelble (ORWK) method. Three different media (water, diiodomethane, and ethylene glycol) were used for the calculations, and all the measurements were performed according to the study [18]. The phenomenon of contact angle hysteresis was also observed in the optical contact angle apparatus using a sessile liquid (water) droplet. After the liquid has advanced over a previously unwetted surface (i.e., when the solid/liquid contact area increases), the maximum contact angle at the three-phase contact line is referred to as the advancing angle ($\theta_{a}$). A minimum contact angle is measured at the contact line when the liquid is retracted over a previously wetted surface (i.e., when the contact area shrinks); this is referred to as a receding angle ($\theta_{r}$). Contact angle hysteresis is defined as the difference between the (maximum) advancing and (minimum) receding angles: ${\Delta \theta }_{\mathrm{hyst}}={\;\theta }_{a}-{\;\theta }_{r}$ [19].

## 3. Results and Discussion

### 3.1. Surface Roughness and Morphology

**Figure 1:**
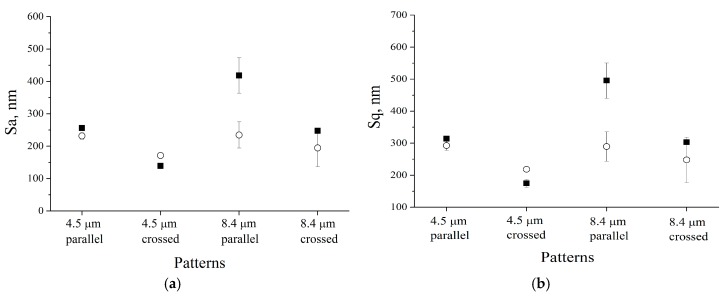
The roughness parameters of different Ti surface patterns with a 35 × 35 ${\mu m}^{2}$ scan area: ■—without hydroxyapatite (HA) film, and ○—with HA film. (**a**) *S_a_* roughness parameter; (**b**) *S_q_* roughness parameter.

To study the changes in the morphology, AFM analysis was carried out on the uncoated and HA-coated patterned Ti substrates. The results presented in Figure 1 reveal that deposition of the HA coating resulted in changes in microscale surface roughness of the initial structured Ti substrate. In general, after HA coating deposition, the roughness parameters *S*_a_ and *S*_q_ decreased. For the grooves with a periodicity of 8.4 μm, the average surface roughness after deposition of the HA coating was lower than that of the structured Ti substrate. This phenomenon may be related to the growth mechanism of the HA coating. During the growth, the HA coating tends to fill the grooves, which results in a surface smoothening effect.

**Figure 2:**
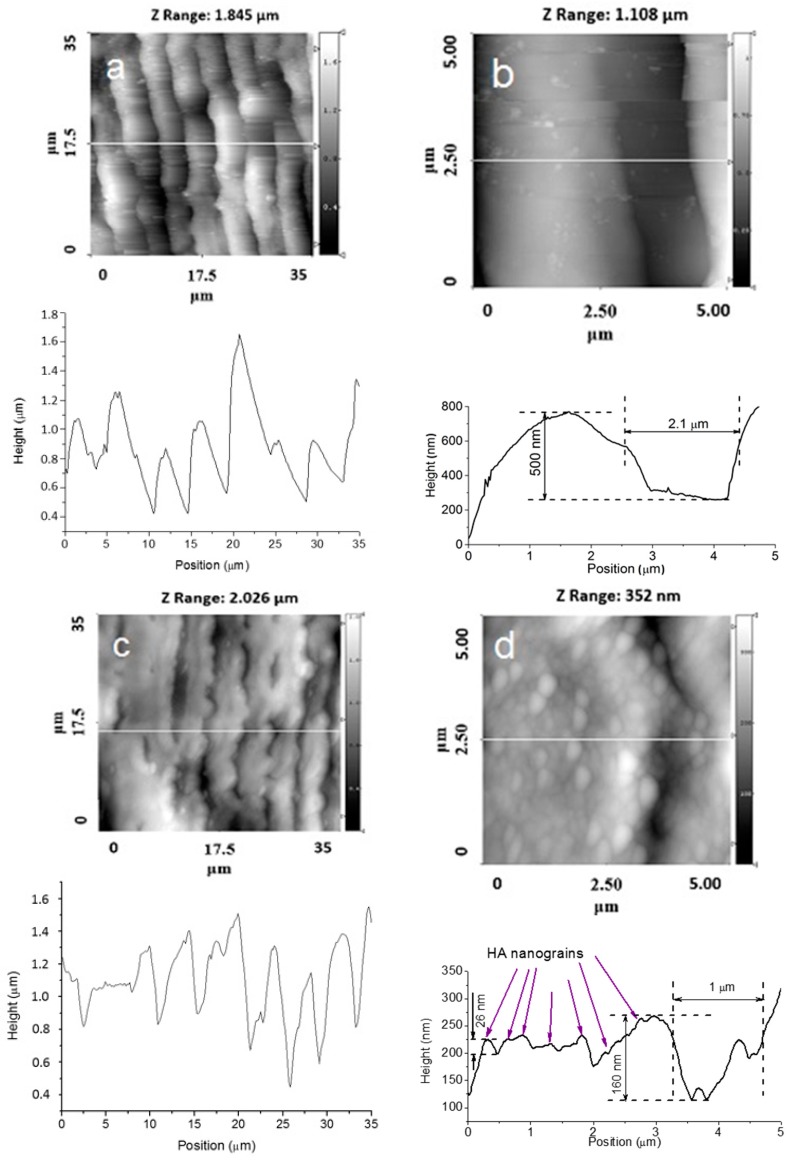
Atomic force microscopy (AFM) images of the surface topographies of Ti samples with parallel grooves and a periodicity of approximately 4.5 µm. (**a**) 35 × 35 ${\mu m}^{2}$; (**b**) 5 × 5 ${\mu m}^{2}$ without a HA film; (**c**) 35 × 35 ${\mu m}^{2}$; and (**d**) 5 × 5 ${\mu m}^{2}$ with a HA film.

**Figure 3:**
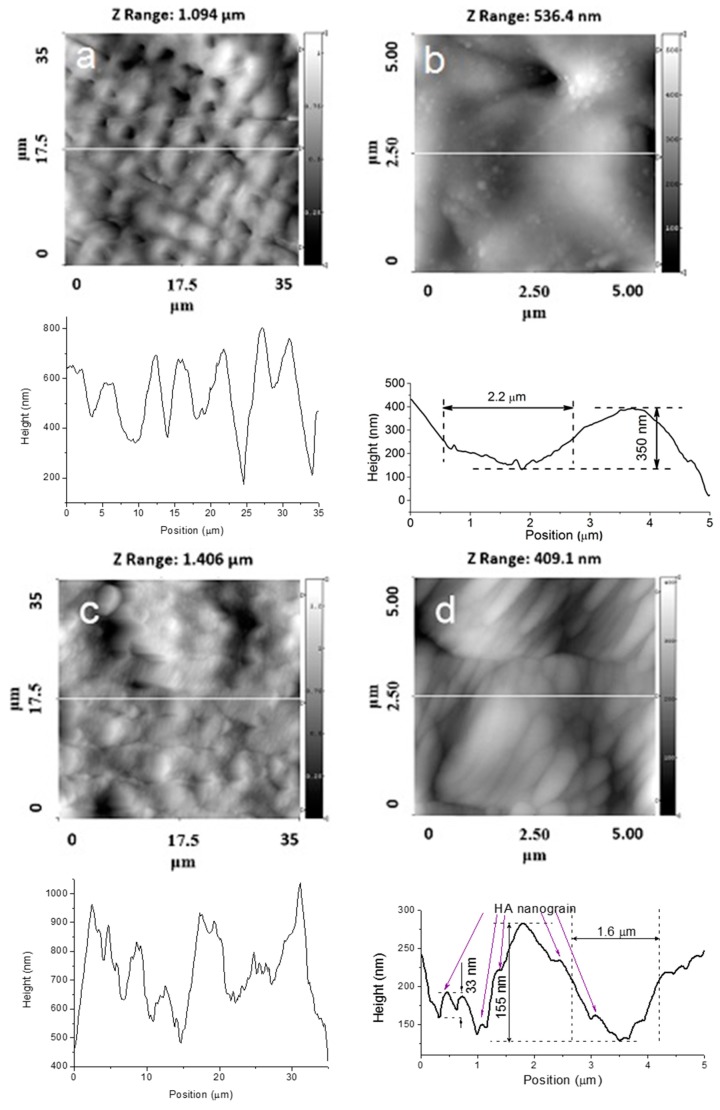
AFM images of the topographies of Ti samples with a crossed periodicity of approximately 4.5 µm. (**a**) 35 × 35 ${\mu m}^{2}$; (**b**) 5 × 5 ${\mu m}^{2}$ without a HA film; (**c**) 35 × 35 ${\mu m}^{2}$; and (**d**) 5 × 5 ${\mu m}^{2}$ with a HA film.

**Figure 4:**
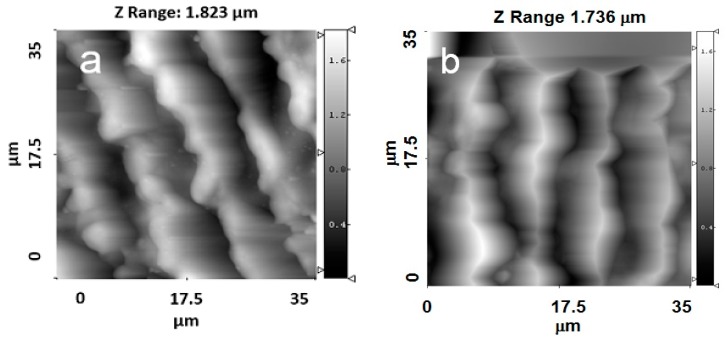
AFM images of the topographies of Ti samples with parallel grooves with a periodicity of approximately 8.4 µm. (**a**) 35 × 35 ${\mu m}^{2}$; (**b**) 35 × 35 ${\mu m}^{2}$ with a HA film.

**Figure 5:**
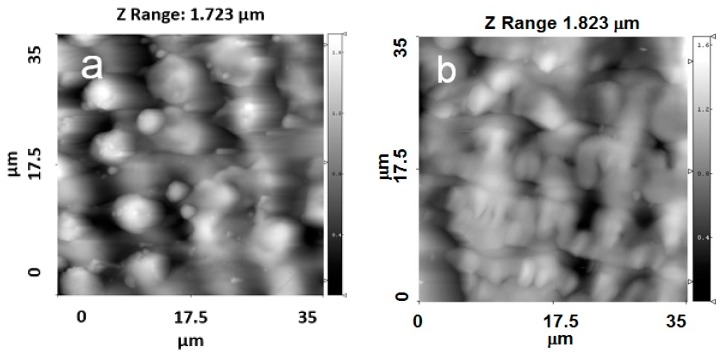
AFM images of the topographies of Ti samples with crossed grooves with a periodicity of approximately 8.4 µm. (**a**) 35 × 35 ${\mu m}^{2}$; (**b**) 35 × 35 ${\mu m}^{2}$ with a HA film.

Figure 2, Figure 3, Figure 4 and Figure 5 show typical AFM images of the HA coating prepared using rf magnetron sputtering on the laser micro-textured Ti surfaces. The microstructure of the surfaces is easily observed using 35 × 35 ${\mu m}^{2}\;$scan areas. For the 5 × 5 µm^2^ scan area, clear grains of the HA coating with definite boundaries can be observed (Figure 2d and Figure 3d). The deposited coating was homogenous and revealed a regular grain-like morphology, which is typical for a thin film deposited by rf magnetron sputtering [20,21]. The surface had a grain-like morphology with a grain size from 0.36 to 0.73 µm in the 5 × 5 ${\mu m}^{2}$ scan area (Figure 2d and Figure 3d). Therefore, the surface of the treated Ti substrates exhibited a multiscale structure.

The surface topography on the microscale, sub-microscale, and nanoscale changed after the HA coating deposition, and this was observed in the AFM profile. Scans obtained for the 35 × 35 µm^2^ scan area showed that changes in the surface topography occurred after the HA coating deposition. During deposition, the film presumably fills the grooves of the Ti substrate. The AFM profile analysis of the HA-coated Ti shows a surface decorated with nanoscale grains (Figure 2d and Figure 3d). The average grain height was approximately 30 nm, which resulted in an increase in the nanoscale roughness of the surface. The most significant changes of the surface topography occurred in surface structured Ti with parallel grooves. For a 5 × 5 µm^2^ scan area, the HA coating resulted in significant shrinkage in the as-prepared grooves, and the structure of the grooves was modified (Figure 2b,d). As shown in Figure 2b, separate grooves with parallel periodicity can be characterized with a full width at half-maximum (FWHM) of ~2.2 µm and an average groove depth of ~500 nm. After the HA coating deposition, the FWHM decreased to ~1.8 µm and the average groove depth reduced to ~200 nm (Figure 2d).

Changes in the surface topography can be observed for the crossed patterns on the Ti surface. Moreover, after deposition of the HA coating, the cross-pattern topography could no longer be clearly observed, as the grooves were filled with the coating material. For the Ti samples with parallel grooves with a periodicity of 8.4 µm, a lower amount of the coating material filled the grooves (Figure 4a,b).

For the Ti samples with crossed grooves with a periodicity of approximately 8.4 µm, the changes in the surface topography were more pronounced than in the grooves with a periodicity of approximately 4.5 µm (Figure 5a,b). The crossed patterns on the Ti surface were still observed after the deposition process.

Topographical features on material surfaces are important to cell and tissue response to biomaterials [22,23,24]. Micro-rough surfaces may stimulate greater bone contact with the material by promoting the production of local osteogenic factors and the expression of differentiation markers. However, the role of nanometer roughness has not been clearly defined [22,23,24].

Previously published results showed that the topography of micro-rough Ti surfaces can significantly influence the attachment and growth of cells [22]. Multidirectional groove designs have been shown to contribute to improved MC3T3-E1 cell adhesion in different directions [24]. Larger microgrooves (greater than ~1 μm in depth) have been proposed to increase plaque uptake in dental applications.

Ulerich et al. investigated the effect of multiscale laser texturing of a Ti6Al4V substrate on the adhesion of osteoblasts [25]. The surface chemical composition can influence cell attachment and reaction to a metal by modifying the adsorption of proteins or by activating different cellular pathways of nearby cells [25]. Unique laser-induced structures can modify the morphology and local chemistry of the surface, which makes it more favourable for cells to grow in certain patterns or to grow at a particular density, depending on the features at various size scales [26].

### 3.2. Phase and Structure Characterization

**Figure 6:**
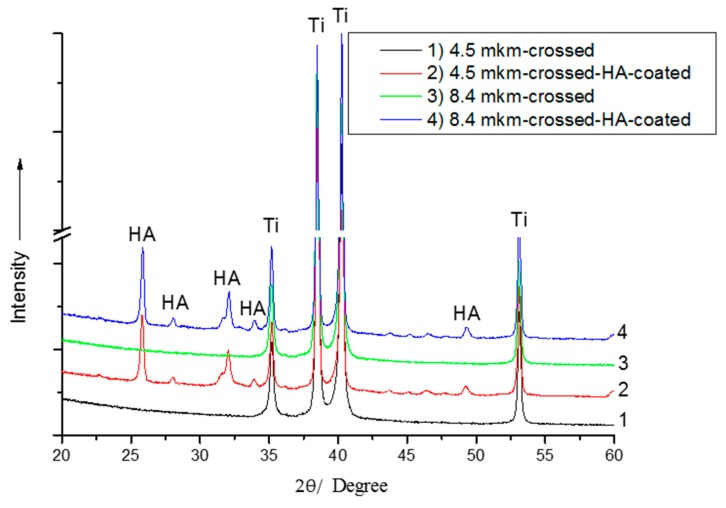
A typical X-ray diffraction (XRD) pattern of HA-coated structured Ti with crossed grooves and a periodicity of 4.5 µm.

The typical XRD patterns of HA-coated patterned Ti substrates with crossed grooves and periodicities of 4.5 µm and 8.4 µm are presented in Figure 6. Only the reflexes attributed to the HA coating are observed, which confirms that the HA film is crystalline and phase-pure. The preferential (002) crystallographic orientation is observed, which is typical for an rf magnetron sputter-deposited thin film [27]. Moreover, the crystallographic orientations of the deposited HA coating can be controlled to either (002) or (300) by modulating the deposition parameters, the thickness of the HA film, and the movement of the substrate during the deposition process [12]. The crystallite size and microstrain determined for the HA coating were 34 ± 1 nm and 0.06%, respectively. In the pattern, only the peaks attributed to the hexagonal α-Ti phase were identified. The lattice parameters of the Ti substrate were determined to be *b* = *a* = 2.9458 and *c* = 4.677 Å. The lattice parameters of the HA coating prepared on the Ti surface were determined as *b* = *a* = 9.4125 and *c* = 6.9167 Å. The crystallite size and microstrain evaluated for the Ti substrate were 42 ± 2 nm and 0.05%, respectively. No significant effect of the Ti surface, which was patterned by the DLIP technique, was observed on the phase composition of either the Ti substrates or the HA-coated structured Ti substrates. Thus, according to the XRD results, no surface TiO_x_ (x < 2) layer was formed through surface processing by the DLIP technique. It has also been reported elsewhere that DLIP processing of the Ti surface does not result in the formation of an oxide layer [7,28]. This is the primary difference between surface processing of Ti by DLIP and conventional heat treatment of Ti in dry air, where Ti is oxidized [29].

### 3.3. Wettability and Surface Free Energy

**Figure 7:**
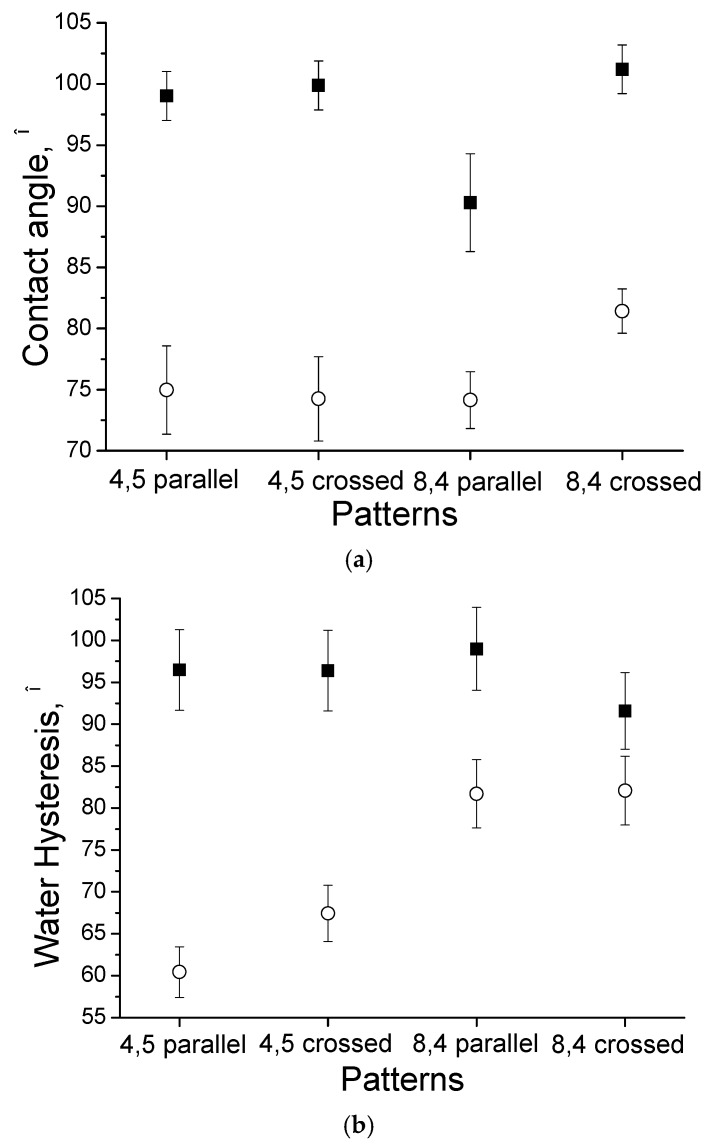
The contact angle (**a**) and water hysteresis; (**b**) measurements for different patterns on the surface of Ti: ■—without HA film, and ○—with HA film.

The evolution of the contact angle and water hysteresis for droplets on the laser-treated uncoated (■) and HA-coated (○) Ti surfaces is presented in Figure 7. The contact angle for the crossed grooves with a periodicity of approximately 8.4 µm is larger than that for the parallel grooves with a periodicity of approximately 8.4 µm for both the HA-coated and uncoated substrates.

The uncoated Ti surface with parallel grooves and a periodicity of approximately 4.5 µm resulted in a high static water CA of 99° ± 2°, which is indicative of surface hydrophobicity. The average water CA for the Ti substrate after the deposition of the HA film was measured as 75° ± 4°, which is associated with surface hydrophilicity. In Figure 6, the water CA hysteresis was determined to be 96.4° ± 1.9° and 67.4° ± 3.4° for the uncoated and HA-coated Ti surfaces, respectively.

The surface wettability of artificial materials is one of the most important factors that determines cell adhesion. Tamada et al. claimed that a surface with a water contact angle of 70° represents the most suitable surface for cell adhesion [30]. In our study, the water contact angle was close to 70° after the deposition of HA. Therefore, the coating can provide beneficial effects for cell adhesion compared with uncoated Ti surfaces. Surface nanotextures—which provide increased surface area and finer surface roughness—may result in improved mechanical interlocking between the tissue and implant [31].

**Figure 8:**
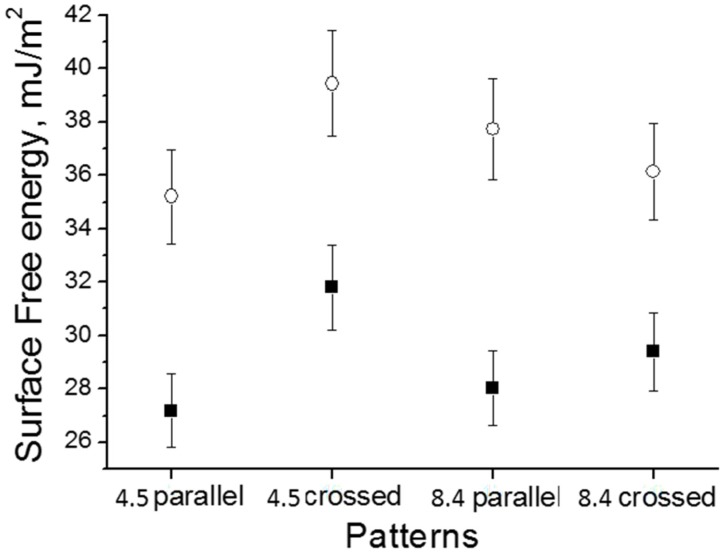
The surface free energy measurements for different patterned Ti surfaces: ■—without a HA film, and ○—with a HA film.

The surface free energy ***σ*** calculations using the CA data indicated that HA-coated Ti surfaces with parallel grooves and a periodicity of approximately 8.4 µm had a significantly larger surface energy than uncoated Ti surfaces (Table 1). The surface free energy of the uncoated substrates was 28.04 mJ/$m^{2}$. Compared with the uncoated Ti surface, HA-coated Ti showed a significantly increased polar component $\sigma_{s}^{p}$ and a larger total surface free energy *σ* (37.73 mJ/$m^{2}$) (Figure 8). The surface free energy is known to have an effect on cell attachment and spreading [32]. The study in [33] demonstrated a strong mutual dependence of cell adhesion and proliferation and platelet activity on the surface free energy of the tested implants, and this was particularly true for polar components. Ti substrates with a low polar component $\sigma_{s}^{p}$ exhibited a very low adhesion shear strength, and the adhesion shear strength of the adhered fibroblasts increased as $\sigma_{s}^{p}$ increased.

The water hysteresis of the uncoated Ti surfaces was larger than that of the HA-coated Ti surfaces. After deposition of the HA coating, Ti surfaces with parallel grooves and a periodicity of approximately 4.5 µm had the lowest water hysteresis (60.40° ± 1.21°). The largest water hysteresis of HA-coated Ti surfaces with crossed grooves and a periodicity of approximately 8.4 µm was 82.09° ± 4.10°. Therefore, the water hysteresis for the hydrophilic surface was less than that for the more hydrophobic Ti surface. In other studies [34,35], it was determined that the magnitude of the hysteresis obtained for the films cast from hydrophobic solvents was less than that for hydrophilic solvents. In addition, the contact-angle hysteresis increased as the contact angle decreased; however, the opposite results were obtained here. The contact angle hysteresis was strongly influenced by the surface roughness and the surface heterogeneity.

The basic correlations between the surface roughness and contact angle hysteresis were originally defined by Wenzel, Cassie, and Baxter [36]. The Cassie–Baxter bridging state represents a non-wetting contact mode where water droplets can roll off easily due to the low contact angle hysteresis. The Wenzel state denotes a wetting contact mode where water droplets can be pinned on the surface to form a high contact angle hysteresis [36]. However, during the culturing time under the conditions used, a partial transition from a Cassie–Baxter state to a Wenzel state may occur [37,38]. In the Wenzel state, the liquid medium is in intimate contact with the solid aspirates, which increases the surface contact area for possible cell contact. In this context, it is worth mentioning a previous controversial discussion on the correlation between the wettability and the water repellence on a cell’s behaviour. Koc et al. presented data that identifies the negative influences of interfacial slip between the liquid and implant surface on protein adsorption [39]. This work indicated that protein adsorption onto superhydrophobic surfaces occurs, and depends on the penetration of the protein solution and available surface area for adsorption. For cells, the aspect of protein adsorption is of utmost importance. An additional related report has been presented by Mano [37], who reported that cells mainly adhered to some points of the aspirates at the surfaces, limiting adhesion and further proliferation.

However, contradictory reports exist regarding the influence of contact angle hysteresis on cell behaviour. As demonstrated in a study by Hsu et al. [40], cell migration on a suitable biomaterial surface may be associated with the ease with which the cell “glides” on the surface. A strong relationship between the sliding angle and cell migration rate was observed. As shown by Hsu and co-workers, a larger cell migration rate correlated with a smaller sliding angle of the substrate. Therefore, the assumption that cell migration could be correlated with the contact angle hysteresis appeared to be reasonable, although the low angle hysteresis describes the ease with which a water droplet rolls off or slides along the surface.

It is known that both surface topography and chemistry influence surface wettability [41]. The roughness parameters measured via AFM revealed that the different rf power levels resulted in changes in the surface roughness parameters (Figure 1). Using the following equation, r = 1 + *S*_dr_/100 (S_dr_—developed interfacial area ratio), it was possible to obtain the values of the roughness factor (r). The following Wenzel equation cosθ_m_ = rcosθ_y_ (θ_m_—measured contact angle, θ_y_—Young contact angle) was used to derive the values of the Young contact angles from the measured values of the contact angles. It is important to notice that the Wenzel equation is based on the assumption that the liquid penetrates into the roughness grooves (Table 2) [42,43]. By considering the calculated and measured values of the Young contact angles (which were very similar), it is possible to conclude that the change in the surface chemistry, which was linked to deposition of the HA coating, played a more significant role in the surface wettability change compared to the surface roughness.

### 3.4. Antibacterial Properties

Additionally, HA coatings have been applied for many decades to enhance the biocompatibility of the surface of an osteointegrated implant [41,44]. Despite mostly successful procedures and the success of HA-coated implants, post-operative infection remains one of the most common and serious complications. Long-term antibacterial properties are desirable in a biomaterial to prevent implant-associated infections. The broad spectrum of antimicrobial effects associated with silver and its compounds against different microbes has been known for centuries [45,46]. This bactericidal activity is best represented by the release of silver ions in a cooperative oxidation process that requires both dissolved oxygen and protons [47,48], with an amount of evidence that silver nanoparticles themselves can damage microbial cell membranes [49,50]. Much less attention is paid on the literature to investigating the surface topography, wettability, or energy effect on biofilm formation or bacterial adhesion [41]. A balance between the effective antimicrobial activity, cytotoxicity, and mechanical stability of the coatings should be achieved.

Bacterial adhesion on surfaces with different surface free energies has been widely studied, and it has been confirmed that the surface free energy can significantly influence bacterial adhesion [18]. A relationship between bacterial adhesion and the surface free energy was reported, and a surface free energy between 23 and 30 mN·m^−1^ was related to the lowest bacterial adhesion [51]. This is consistent with other reported results. For example, a minimum *Escherichia coli* adhesion was reported for the surface free energy range between 21 and 29 mN·m^−1^ [18]. Boyd et al. found that enhanced adhesion of *S. aureus* occurred on rougher stainless steel compared to smooth surfaces. Surfaces with features on the same scale as the *S. aureus* cells (1 µm) appeared to promote the strongest attachment, due to maximal cell–substrate contact area [52,53]. Whitehead et al. also observed similar trends for bacterial species of different sizes [54]. On the other hand, bacteria have been found to colonize smooth surfaces, such as electropolished stainless steel [55].

It is reported that superhydrophobic surfaces promote the reduction of bacterial adhesion [18]. The presented results revealed that all of the investigated microtopographies provoked a significant reduction (30%–45%) in bacterial adhesion relative to the smooth control samples, regardless of the surface hydrophobicity/hydrophilicity [56]. Cell (*Escherichia coli*) cluster formation per unit area on 5 μm wide line patterns was reduced by 14-fold compared to flat poly(dimethylsiloxane) [57]. When the surface chemistry was the same, the nanorough surface had higher surface energy than the smooth surface of Ti and showed lower bacterial colonization [58].

Thus, macroscopic grooves fabricated on the surface of Ti substrates using the DLIP technique can provide a preferential site for the deposition of bacteria within the valleys, while the microscopic and nanoroughness of the valleys coated with nanostructured HA coating will determine the actual interaction between the surface and bacteria, which should prevent undesired bacterial adhesion in the developed patterned substrates.

## 4. Conclusions

In this study, investigations of the surface microstructure of Ti surfaces produced by the DLIP technique were performed. To generate multiscale structures on microscale Ti surfaces, rf magnetron sputter deposition of ultrathin nanostructured HA films was used to form an additional nanoscale grain morphology. The three-dimensional roughness parameters *S*_a_ and *S*_q_ tended to decrease after deposition of the HA coating. The grain-like morphology of the surface with an average grain size from 0.36 to 0.73 µm for a 5 × 5 ${\mu m}^{2}$ scan area was described. Uncoated Ti surfaces with parallel grooves and a periodicity of 4.5 µm resulted in a static water contact angle of 99° ± 2°, which confirmed the hydrophobic nature of the surface. An average value of the water contact angle for the Ti substrate after the fabrication of the HA film was measured as 75° ± 4°, demonstrating the hydrophilic nature of the surface. In this study, a moderately hydrophilic surface with a contact angle close to 70° was obtained for HA-coated substrates. A significant increase in the polar component and total surface free energy *σ* (37.73 mJ/$m^{2}$) for the HA-coated Ti surface compared to the uncoated Ti surface (28.04 mJ/$m^{2}$) was demonstrated. Therefore, the significance of the obtained results is that different topographical surface features and different surface chemical compositions can be obtained, which can significantly affect cell attachment, proliferation, and differentiation by changing the way that proteins adsorb or by activating different cellular pathways of nearby cells.

## Figures and Tables

**Table 1:** The surface wettability parameters of uncoated structured Ti and HА-coated structured Ti.

Parameters	Patterns							
	4.5 µm Parallel Grooves		4.5 µm Crossed Grooves		8.4 µm Parallel Grooves		8.4 µm Crossed Grooves	
	Uncoated	Coated	Uncoated	Coated	Uncoated	Coated	Uncoated	Coated
$\sigma_{s}^{p}$, mJ/$m^{2}$	0.34 ± 0.02	6.20 ± 0.12	0.12 ± 0.01	5.40 ± 0.11	1.74 ± 0.03	5.94 ± 0.30	0.13 ± 0.01	3.19 ± 0.16
$\sigma_{s}^{d}$, mJ/$m^{2}$	26.84 ± 1.34	29.01 ± 0.58	30.96 ± 0.62	34.04 ± 0.68	26.31 ± 0.53	34.79 ± 1.74	29.27 ± 0.59	32.93 ± 1.65
σ, mJ/$m^{2}$	27.19 ± 1.36	35.21 ± 0.7	31.8 ± 0.64	39.44 ± 0.79	28.04 ± 0.56	37.73 ± 1.89	29.4 ± 0.59	36.13 ± 1.81

**Table 2:** Summary of the θ_m_ and θ_y_ calculated for a 35 × 35 ${\mu m}^{2}$ scan area.

Surface/Parameter	S_dr_, %	Roughness Factor, r	θ_m_, °	θ_y_, °
Etched Ti (unpatterned)	3.32	1.0332	85.2 ± 2	85.6
Uncoated patterned Ti surfaces				
4.5 μm line	11.3 ± 1.3	1.113	98.4 ± 2	98.6
4.5 μm crossed	2.4 ± 0.2	1.024	99.3 ± 2	99.3
8.4 μm line	5.5 ± 0.4	1.055	90.3 ± 4	90.8
8.4 μm crossed	5.3 ± 0.7	1.053	101.2 ± 2	101.2
HA coated Ti patterned surfaces				
4.5 μm line	6.7 ± 0.2	1.067	75.1 ± 2	76.6
4.5 μm crossed	2.5 ± 0.3	1.025	74.1 ± 2	74.7
8.4 μm line	5.2 ± 0.4	1.052	73.5 ± 2	74.9
8.4 μm crossed	2.0 ± 0.4	1.020	81.5 ± 2	81.9
